# Reducing the length of hospital stay for patients undergoing primary total knee arthroplasty by application of enhanced recovery after surgery (ERAS) pathway: a multicenter, prospective, randomized controlled trial

**DOI:** 10.1186/s40001-025-02647-8

**Published:** 2025-05-14

**Authors:** Chenxi Liao, Xingning Lai, Jie Zhong, Wencong Zeng, Jiannan Zhang, Wanxin Deng, Jiayun Shu, Haobo Zhong, Liangyu Cai, Ren Liao

**Affiliations:** 1 Department of Anesthesia and Operation Center, Research Unit for Perioperative Stress Assessment and Clinical Decision, Chinese Academy of Medical Sciences (2018RU012), West China Hospital of Sichuan University, No.37 Guo Xue Lane, Chengdu, 610041 China; 2 Department of Anesthesiology, Huizhou First Hospital, No.20 Jiangbei Sanxin South Road, Huizhou, 516001 China; 3 Department of Anesthesiology, No. 8, Wuxi Traditional Chinese Medicine Hospital Affiliated to Nanjing University of Chinese Medicine, Zhongnan West Road, Wuxi, 214071 China; 4 Department of Anesthesiology, The First People’s Hospital of Longquanyi District, Chengdu, 610100 China; 5 Department of Anesthesiology, Xindu District People’s Hospital of Chengdu, No.199 Yuying Road South Section, Chengdu, 610599 China; 6 Department of Orthopedics, Huizhou First Hospital, No.20 Jiangbei Sanxin South Road, Huizhou, 516001 China

**Keywords:** Primary total knee arthroplasty, Length of hospital stay, Enhanced recovery after surgery

## Abstract

**Background:**

The proportion of elderly patients undergoing Total knee arthroplasty (TKA) is growing. Optimizing and accelerating postoperative recovery for TKA patients is critical in clinical practice. Enhanced Recovery After Surgery (ERAS) is a protocol involving a series of evidence-based perioperative optimization strategies to minimize surgical stress and expedite recovery, and a multidisciplinary ERAS pathway was established jointly by anesthesiologists and orthopedic surgeons in this study. The authors hypothesized that application of the ERAS pathway can reduce the length of hospital stay (LOS) for patients undergoing primary TKA.

**Materials and Methods:**

This multicenter, prospective, randomized controlled trial was conducted from February 1, 2021 to January 31, 2023, and included patients undergoing elective primary TKA. 320 patients were randomly assigned to either the ERAS group (practice according to the ERAS pathway) or the control group (without ERAS pathway implementation) in a 1:1 ratio. The primary outcome was the total LOS in hospital.

**Results:**

LOS in the ERAS group was 5.92 ± 1.16 days, significantly shorter than the 8.17 ± 1.76 days in the control group (p < 0.001). Postoperative LOS and time to independent ambulation were significantly shorter in the ERAS group compared to the control group (p < 0.001). On postoperative day 1, significantly less participants reported pain both in rest and during mobilization in the ERAS group than the control group (p < 0.001). The incidences of thirst and postoperative nausea and vomiting (PONV) was significantly reduced in the ERAS group compared to the control group (16.8% vs. 88.6%, and 2.6% vs. 24.7%, respectively, p < 0.001). No perioperative deaths or reoperations within 30 days occurred in either group.

**Conclusion:**

The application of an ERAS pathway for primary TKA significantly reduces LOS, alleviates postoperative pain, and lowers the incidence of adverse events compared to perioperative management without ERAS pathway implementation.

*Trial registration*: The National Institutes of Health Clinical Trials Registry, NCT03517098. Registered on April 24, 2018.

## Highlights

- An ERAS pathway consisting of optimized fasting, minimization of long-acting opioids, use of tranexamic acid and dexamethasone, optional use of a tourniquet, avoidance of urinary catheter and surgical drains, and local anesthetics surgical wound infiltration can reduce the length of hospital stay, which reflects the early recovery after surgery for patients undergoing primary TKA.
- The incidences of adverse events such as thirsty, PONV, and postoperative pain both in rest and during mobilization can be reduced by application of ERAS pathway.
- This multicenter, prospective, randomized controlled trial provides a robust evidence regarding the clinical safety and efficacy of ERAS pathway for primary TKA.

## Introduction

Total knee arthroplasty (TKA) is an effective treatment for end-stage knee osteoarthritis. With the progression of an aging society worldwide, the number of TKA has been steadily increasing. Approximately 700,000 TKA procedures were performed annually in the United States, and this number is anticipated to increase to 3.48 million by 2030 [1]. Furthermore, the proportion of elderly patients undergoing TKA is growing, with individuals aged 65 years and older accounting for up to 70% of cases [2]. Geriatric patients undergoing TKA often present with comorbid conditions such as hypertension, coronary artery disease, diabetes, and pulmonary dysfunction, while also managing long-term medication use for osteoarthritis or rheumatoid arthritis [3]. These factors complicate perioperative management for both surgeons and anesthesiologists, posing additional logistical challenges. As a result, these complexities increase the burden on healthcare providers and escalate societal healthcare costs. To address these issues effectively, optimizing and accelerating postoperative recovery for TKA patients has become a priority in clinical practice and public health [1–3].

Enhanced Recovery After Surgery (ERAS) is a multidisciplinary approach involving a series of evidence-based perioperative optimization strategies to minimize surgical stress and expedite recovery [4]. ERAS protocols have demonstrated synergistic effects in reducing perioperative complications, shortening hospital stays, and improving patient satisfaction in various surgical fields, including colorectal, thoracic, and orthopedic surgeries [5–8]. The participation of anesthesiologists is a crucial component of ERAS implementation, as maintaining intraoperative hemodynamic stability is key to reducing postoperative complications and promoting rapid recovery. Accordingly, the choice of anesthetic agents is vital during the maintenance phase of anesthesia within an ERAS framework. Previous studies have consistently recommended regional anesthesia techniques such as epidural anesthesia, spinal anesthesia, or nerve blocks as part of ERAS protocols, primarily because these approaches theoretically reduce dependency on cardiopulmonary or renal function compared to general anesthesia [4–6, 9]. However, regional anesthesia and nerve blocks may impede motor function, delaying independent ambulation and functional recovery. Furthermore, regional anesthesia often necessitates urinary catheterization, which is not conducive to accelerated recovery [10, 11]. Thus, for surgeries like TKA, which are characterized by relatively short durations (< 2 h) and minimal blood loss (< 400 ml) and demand prompt restoration of muscle strength and mobility, innovative ERAS protocols are needed to avoid catheterization and minimize motor function impairment.

Advances in anesthetic pharmacology have introduced short-acting general anesthetic and analgesic agents such as propofol, desflurane and remifentanil that ensure both safety and efficacy with rational and accurate application. For example, desflurane, an inhalational anesthetic, exhibits a low blood-gas partition coefficient, facilitating rapid induction and recovery, rapid metabolism, minimal accumulation, and minimal hemodynamic disturbance. Desflurane does not adversely affect critical organ functions, such as the heart, lungs, or kidneys [12]. Moreover, the short-acting anesthetics will not cause the weakened muscle strength and not affect the patient’s early ambulation abilities after metabolized in a short period to facilitate the early recovery. Given these pharmacological characteristics, we proposed a general anesthesia-based ERAS pathway combined with the use of short-acting anesthetic agents and other improvement of perioperative processes including shortened fasting time, use of dexamethasone and tranexamic acid, no indwelling urine catheters, no tourniquet used, no drainage tube after surgery, and early mobilization. We hypothesized that application of the ERAS pathway can reduce the length of hospital stay (LOS) for patients undergoing primary TKA, which represented the early recovery after surgery.

We aimed to establish a multidisciplinary ERAS team led jointly by anesthesiologists and orthopedic surgeons to develop a nationwide ERAS pathway for primary TKA in our study, and to provide robust evidence regarding the clinical safety and efficacy of a general anesthesia-based ERAS pathway for primary TKA.

## Materials and methods

### Study aim, design and settings

This study is a multicenter, prospective, randomized controlled clinical trial designed to test the superiority of the general anesthesia-based ERAS pathway in term of the total hospital length of stay (LOS) compared with the current clinical practice for patients undergoing TKA, conducted from February 1, 2021 to January 31, 2023 at West China Hospital of Sichuan University, Huizhou First Hospital, Wuxi Traditional Chinese Medicine Hospital Affiliated to Nanjing University of Chinese Medicine, the First People’s Hospital of Longquanyi District, and Xindu District People's Hospital of Chengdu. Ethical approval was obtained from the Biomedical Ethics Committee of West China Hospital, Sichuan University (Approval Number: 2018–82) on February 18, 2019, and the trial was registered at http://www.clinicaltrials.gov (Registration Number: NCT03517098). It was conducted under the regulations of the Helsinki Declaration. Written informed consent was obtained from all participants or their legal guardians before enrollment.

### Study population

Participants were screened for participation by a team of anesthesiologists and orthopedic surgeons, and eligible participants were visited by investigators one day before surgery. The investigators explained the written informed consent in detail and answered all the queries regarding the study, and participants were taught the way to evaluate the numerical rating scales (NRS) scores and the concept of postoperative ambulation and exercise.

### Inclusion criteria

- Patients scheduled for primary TKA;
- Aged > 18 years, with both genders;
- Signed written informed consent.

### Exclusion criteria

- Pregnant or lactating women;
- Personal or family history of malignant hyperthermia;
- Known allergy to desflurane or other anesthetic agents;
- History of substance abuse;
- Cognitive or communication impairments;
- Participation in other clinical trials;
- Unwillingness or inability to comply with the study protocol.

### Withdrawal criteria

- ﻿Voluntary withdrawal;
- Development of exclusion criteria during the study;
- Safety was compromised.

### Randomization and blinding

The central randomization system (CRS) was used to screen and randomize the participants with a 1:1 allocation ratio. Five centers were included and stratified first to guarantee the balance between two groups within each center. Participants were enrolled sequentially, and their screening numbers and relevant information were entered into CRS. A computer-generated randomization number and group allocation, pre-programmed on the online CRS platform, was retrieved. This was an open-label trial due to the different processes during hospitalization, the participants, investigators, research assistants, and physicians were aware of the group allocation, while follow-up staff, and statisticians remain blinded to the treatment assignment.

### Procedures

#### Group interventions

##### ERAS Group

Participants in the ERAS group were managed according to the following protocols:

### Orthopedic management

- Strict adherence to fasting guidelines: 8 h for protein and fats, 6 h for starches, and 200 ml of clear fluids 2 h before surgery [13].
- Administration of tranexamic acid (20 mg/kg) 15 min before skin incision.
- No urinary catheter placement.
- Optional or minimal use of a tourniquet during critical steps only.
- No placement of surgical drains.
- Initiation of low-molecular-weight heparin 6 h postoperatively.

### Anesthesia management

- Monitoring parameters: electrocardiogram, pulse oximetry, non-invasive blood pressure, and bispectral index (BIS).
- Administration of intravenous 10 mg of dexamethasone prior to anesthetic induction.
- Anesthetic induction with sufentanil (0.2 µg/kg), cisatracurium (0.15 mg/kg), and propofol (1 mg/kg), with laryngeal mask airway placement when the BIS value decreased to 50.
- Maintenance anesthesia with 5–7% desflurane inhalation and continuous infusion of remifentanil (0.15–0.3 µg/kg/min), maintaining BIS values between 40 and 60.
- Surgical wound infiltration with 40–50 ml of 0.2% ropivacaine before the end of surgery.
- No postoperative patient-controlled analgesia (PCA) pumps were used.

### *ERAS group*

Participants in the control group were managed according to the routine clinical practices of each participating center. No standardized protocols were imposed regarding preoperative management, choice of anesthesia or analgesia, dietary intake, urinary or surgical drain placement, or tourniquet use.

Regardless of group allocation, clinical decisions regarding catheterization, nasogastric or surgical drain placement, or tourniquet use were made at the discretion of the attending physician based on the participant’s clinical condition. For example, if a participant in the ERAS group developed postoperative urinary retention, a urinary catheter may be placed at the physician's discretion. Such participants would remain in the study, and their data were analyzed as part of the ERAS group.

### Postoperative pain management

For patients reporting significant pain (NRS > 4), the attending physician administered rescue analgesics according to routine clinical practice. No restrictions were placed on the choice or dosage of analgesics.

### Outcome measures

#### Primary outcome

The primary outcome was the total length of hospital stay (LOS), defined as the duration from hospital admission to the day of discharge (measured in days).

#### Secondary outcomes

1. Postoperative LOS: defined as the duration from the day of surgery to the day of discharge (measured in days).
2. 30-day all-cause mortality.
3. Incidence of complications during hospitalization, categorized into five grades based on severity:
  Grade I: Resolved with temporary treatment, such as postoperative nausea and vomiting (PONV).
  Grade II: Extended hospital stay, such as pneumonia requiring antibiotic treatment.
  Grade III: Life-threatening events requiring interventions, with recovery achieved during hospitalization, such as postoperative bleeding requiring surgery.
  Grade IV: Long-term harm persisting beyond 30 days postoperatively, with significant decline in quality of life, such as acute myocardial infarction.
  Grade V: Death occurring within 30 days postoperatively, which is also recorded as a separate secondary outcome.
4. Time to independent ambulation: defined as the duration from the end of surgery to the time when the participant can walk independently for at least 10 m without assistance (walking aids such as crutches are permitted, measured in hours).
5. Resting NRS pain score on postoperative day 1: graded as 0 (no pain), 1–3 (mild pain), 4–6 (moderate pain), or 7–10 (severe pain).
6. Movement-related NRS pain score on postoperative day 1: graded similarly as resting pain scores (0 = no pain, 1–3 = mild pain, 4–6 = moderate pain, 7–10 = severe pain).
7. Proportion of participants requiring postoperative analgesia.
8. Incidence of hospital re-opertion within 30 days postoperatively.

### Statistical analysis

Statistical analysis was performed using SPSS 18.0 software (Statistic Package for Social Science, SPSS, Inc., Chicago, IL, USA), applying an intent-to-treat (ITT) principle for both primary and secondary outcomes. Missing values in secondary outcomes were imputed using either the last observation carried forward (LOCF) method or, in cases where no prior observation was available, by group-specific mean or median substitution. Continuous variables were tested for normality. For normally distributed data, such as total LOS and postoperative hospital stay, results were expressed as mean ± standard deviation, and group comparisons were conducted using the independent sample t-test. Non-normally distributed data were reported as medians (minimum, maximum), with comparisons between groups analyzed using the rank-sum test. Categorical variables, such as gender, American Society of Anesthesiologists (ASA) classification, or incidence of complications during hospitalization, were expressed as [n/n or n/n (%)] and analyzed using the Chi-square test or Fisher’s exact test. A *P* < 0.05 was considered statistically significant.

### Theory/calculation

The hypothesis is that the ERAS pathway would reduce LOS compared to the current clinical practice. Based on retrospective data from West China Hospital of Sichuan University, the LOS for the ERAS group is estimated to be 4.9 ± 1.6 days, compared to 5.3 ± 1.6 days for the control group. Using a two-sided significance level (α) of 0.05 and a power (1-β) of 0.8, a sample size of 132 participants per group was required. Accounting for a 20% dropout rate, each group will include 160 participants, for a total of 320 participants [14].

## Results

**Fig. 1:**
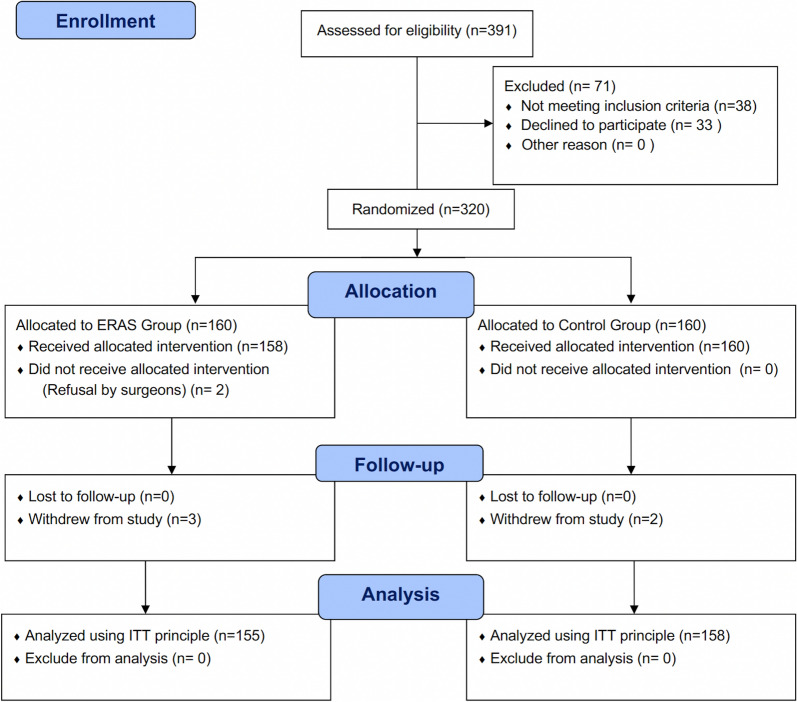
CONSORT flow diagram

A total of 391 participants were screened for this study at West China Hospital of Sichuan University, Huizhou First People’s Hospital, and Wuxi Affiliated Hospital of Nanjing University of Chinese Medicine, from February 1,2021 to January 31, 2023. Out of the 391 participants, 38 participants did not meet at least one item of the inclusion criteria and 33 declined to participate. Finally, 320 participants were enrolled and randomized. In the ERAS group, 158 participants were assigned and received the intervention, and 2 participants did not undergo the intervention due to refusal by their surgeons. All 160 participants in the control group received their respective interventions. During the follow-up, 3 participants in the ERAS group withdrew from the study, and 2 participants in the control group withdrew from the study. Therefore, the final analysis included 155 participants in the ERAS group and 158 participants in the control group. A ﻿CONSORT flow diagram is illustrated in Fig. 1.

### Baseline characteristics

There were no significant differences between the two groups in baseline characteristics including age, gender, height, weight, body mass index (BMI), ASA classification, and preoperative comorbidities (Table 1).

**Table 1:** Baseline characteristics

Characteristics	ERAS Group (n = 155)	Control Group (n = 158)	*P*
Age (years)^a^	66.73 ± 7.22	66.63 ± 9.20	0.913
Gender (Female/Male)^b^	112/43	117/41	0.799
Height (cm)^a^	162.12 ± 10.29	164.16 ± 10.09	0.064
Weight (kg)^a^	65.64 ± 7.22	66.73 ± 7.22	0.200
BMI^a^	24.95 ± 3.44	24.78 ± 3.13	0.641
Preoperative Hemoglobin (Hb, g/L)^a^	11.73 ± 1.95	11.42 ± 1.67	0.122
ASA Classification (II/III)^b^	51/104	54/104	0.905
Hypertension [Yes/No (%)]^b^	111/44 (71.6%)	118/40 (74.1%)	0.610
Diabetes [Yes/No (%)]^b^	117/38 (75.5%)	114/44 (72.2%)	0.523
Coronary Artery Disease [Yes/No (%)]^b^	32/123 (20.6%)	27/131 (17.1%)	0.471
Chronic Obstructive Pulmonary Disease (COPD) [Yes/No (%)]^b^	28/127 (18.1%)	35/123 (22.2%)	0.399

^a^Means ± SD compared by group independent sample t-test

^b^[n (%)] compared using χ2 test or Fisher’s exact test

### Surgical and anesthetic variables

No significant differences were found in terms of anesthesia duration, surgical duration, or intraoperative remifentanil consumption between the two groups. The intraoperative sufentanil dosage was 14.80 ± 3.50 μg in the ERAS group and 25.38 ± 7.48 μg in the control group (p < 0.001, Table 2). Tourniquets were applied for 4 participants in the ERAS group, whereas all participants in the control group did so. Four participants in the ERAS group had indwelling urinary catheters compared to 130 participants in the control group. Two participants in the ERAS group had surgical drains, in contrast to 130 participants in the control group. These three parameters all showed significant differences between the groups (p < 0.001, Table 2). The volume of intraoperative fluid administration was significantly lower in the ERAS group compared to the control group (p < 0.05, Table 2). Regarding perioperative blood transfusions, no participants in the ERAS group required a transfusion, which was significantly fewer than the 7 participants in the control group who required transfusions (p < 0.05, Table 2).

**Table 2:** Surgical and anesthetic variables

Variables	ERAS Group (n = 155)	Control Group (n = 158)	*P*
Anesthesia duration (min)^a^	120.26 ± 16.74	118.78 ± 19.63	0.474
Surgical duration (min)^a^	86.67 ± 12.77	86.13 ± 16.05	0.746
Sufentanil dosage (µg)^a^	14.80 ± 3.50	25.38 ± 7.48	0.000^d^
Remifentanil dosage (mg)^a^	1.18 ± 0.73	1.08 ± 0.47	0.093
Intraoperative fluid volume (ml)^a^	862.26 ± 176.85	1219.62 ± 274.14	0.000^d^
Tourniquet use [Yes/No (%)]^b^	4/151 (2.6%)	158/0 (100%)	0.000^d^
Urinary catheterization [Yes/No (%)]^b^	4/151 (2.6%)	130/28 (82.3%)	0.000^d^
Drain placement [Yes/No (%)]^b^	2/153 (1.3%)	130/28 (82.3%)	0.000^d^
Perioperative transfusion [Yes/No (%)]^b^	0/155 (0%)	7/151 (4.4%)	0.015^c^

^a^Means ± SD compared by group independent sample t-test

^b^[n (%)] compared using χ2 test or Fisher’s exact test

^c^*P* < 0.05

^d^*P* < 0.001

### Primary outcome

LOS for participants in the ERAS group was 5.92 ± 1.16 days, significantly shorter than the 8.17 ± 1.76 days in the control group, representing a reduction of approximately 2 days (p < 0.001, Table 3).

**Table 3:** Postoperative variables

Characteristics	ERAS Group (n = 155)	Control Group (n = 158)	*P*
Primary outcome LOS (days)	5.92 ± 1.16	8.17 ± 1.76	0.000^c^
Secondary outcomes			
Postoperative LOS (days)^a^	4.06 ± 1.09	6.06 ± 1.87	0.000^c^
Time to independent ambulation (hours)^a^	5.02 ± 1.76	6.30 ± 1.80	0.000^c^
Day 1 resting pain NRS score [None/Mild/Moderate (%)]^b^	115/37/3 (74.2%/23.9%/1.9%)	71/72/15 (44.9%/45.6%/9.5%)	0.000^c^
Day 1 movement pain NRS score [None/Mild/Moderate/Severe (%)]^b^	44/64/47/0 (28.4%/41.2%/30.4%/0%)	17/38/97/6 (10.8%/24.1%/61.4%/3.8%)	0.000^c^
Postoperative analgesia [Yes/No (%)]^b^	40/115 (25.8%)	76/82 (48.1%)	0.000^c^
Thirst [Yes/No (%)]^b^	26/129 (16.8%)	140/158 (88.6%)	0.000^c^
Postoperative nausea and vomiting [Yes/No (%)]^b^	4/151 (2.6%)	39/119 (24.7%)	0.000^c^
Postoperative complications [Yes/No (%)]^b^	6/149 (3.9%)	11/146 (7.0%)	0.290

^a^Means ± SD compared by group independent sample t-test

^b^[n (%)] compared using χ2 test or Fisher’s exact test

^c^*P* < 0.001

### Secondary outcomes

Postoperative LOS and time to independent ambulation were significantly shorter in the ERAS group compared to the control group (p < 0.001, Table 3). On postoperative day 1, 115 participants (74.2%) in the ERAS group reported no resting pain, 37 reported mild pain, and 3 reported moderate pain. In contrast, 71 participants (44.9%) in the control group reported no resting pain, 72 reported mild pain, and 15 reported moderate pain (p < 0.001, Table 3). During mobilization, 44 participants in the ERAS group experienced no pain, 64 reported mild pain, and 47 reported moderate pain, with no cases of severe pain. The incidence of moderate to severe pain in the ERAS group was 30.4%. In the control group, 17 participants experienced no pain, 38 reported mild pain, 97 reported moderate pain, and 6 reported severe pain, with a moderate to severe pain incidence of 65.2% (p < 0.001, Table 3).

The incidences of thirst and postoperative nausea and vomiting (PONV) was significantly reduced in the ERAS group compared to the control group (16.8% vs. 88.6, and 2.6% vs. 24.7%, respectively, p < 0.001, Table 3). No perioperative deaths or reoperations within 30 days occurred in either group. In the ERAS group, 6 participants experienced grade II postoperative complications, including 3 cases of postoperative hypotension, 1 case of diarrhea, and 2 cases of upper respiratory tract infections. In the control group, 11 participants experienced grade II postoperative complications, including 5 cases of postoperative hypotension, 2 cases of postoperative hypertension, and 4 cases of upper respiratory tract infections. All cases were resolved with treatment, and there was no significant difference between the groups (Table 3).

## Discussion

To the best of our knowledge, this is the first prospective, multicenter, randomized controlled trial to test the superiority of ERAS pathway compared to the clinical practice without explicit implementation of ERAS in TKA. The primary result is that application of ERAS pathway can reduce the LOS for patients undergoing primary TKA, which reflects the early recovery for patients could be get by implementing the ERAS pathway. Numerous publications, including clinical studies [15–17], reviews [8, 18, 19], and meta-analyses [6, 20, 21], have demonstrated that compared to conventional management, ERAS protocols were associated with shorter hospital stays, reduced morbidity, decreased stress response, lower healthcare costs, and accelerated perioperative recovery across various types of surgeries. A multicenter study conducted across three hospitals in Victoria reported a significant reduction in LOS following ERAS implementation in elective joint arthroplasty with improved rates of discharge readiness by postoperative Day 3 (59% vs. 41%, *p* < 0.001) and enhanced recovery indicators including earlier ambulation and better pain control [15]. Similarly, in gynecologic surgery, ERAS adoption in abdominal hysterectomy reduced LOS from 2.6 to 2.3 days without increased complication [16], the magnitude of LOS reduction observed in our study is comparable to the 30–50% range cited in broader ERAS literature reviews [19]. This supports the consistency of ERAS effects across surgical disciplines. Another systematic review concerning total joint arthroplasty showed that ERAS likely reduced the LOS after primary elective total hip arthroplasty and TKA, but the study heterogeneity and high risk of bias limited the quality of evidence, and high-quality research is needed to verify the impact of ERAS on total joint arthroplasty [22]. Our study provided a sufficient evidence and helpful supplement to the beneficial effect of multidisciplinary ERAS pathway on TKA. Additionally, a multicenter design was employed to strengthen the clinical relevance and generalizability of our findings. Conducting the trial in various surgical practices and perioperative care further demonstrates that the ERAS pathway can be effectively implemented in diverse hospital environments and across distinct regional healthcare practices in China.

Our results also indicated that the incidence of thirst was significantly lower in the ERAS group compared to the control group (16.8% vs. 88.6%, p < 0.001, Table 3). Similarly, PONV rates were markedly reduced in the ERAS group (2.6% vs. 24.7%, p < 0.001, Table 3). Perioperative thirst and PONV are common clinical issues that negatively impact patient satisfaction with medical care [23]. Traditionally, preoperative fasting for at least 8 h was recommended to maintain an empty stomach and prevent aspiration during anesthesia. However, prolonged fasting often necessitates large intraoperative fluid replacement to counteract hypovolemia, resulting in discomfort due to thirst and adverse outcomes like gastrointestinal hypomotility or pulmonary interstitial edema. In this study, the ERAS pathway followed updated fasting guidelines [13], allowing clear liquids up to 2 h before surgery. This approach maintained perioperative normovolemia, mitigated thirst-related discomfort, and facilitated restrictive fluid management, leading to significantly reduced intraoperative fluid administration in the ERAS group. PONV is influenced by multiple factors, including female sex, history of motion sickness, prolonged fasting, and the use of long-acting opioids [24, 25]. The ERAS pathway addressed these risk factors through shortened fasting times, reduced reliance on long-acting opioids, and administration of dexamethasone preoperatively. These multimodal preventive and therapeutic measures resulted in a significantly lower PONV incidence in the ERAS group compared to the control group.

Pain is a critical determinant of postoperative functional recovery. While opioids are among the most effective agents for postoperative analgesia, they are also associated with adverse effects such as urinary retention, respiratory depression, gastrointestinal hypomotility, increased PONV risk, and, in elderly patients, potential postoperative cognitive dysfunction (POCD), which can impair long-term outcomes [24–26]. In this study, the ERAS pathway focused on minimizing long-acting opioid use by employing short-acting agents such as remifentanil, and desflurane [27], in combination with 0.2% ropivacaine for layer-by-layer local infiltration anesthesia at the surgical site. This strategy prevented rebound pain following discontinuation of remifentanil, reduced opioid-related adverse effects, and lowered the consumption of long-acting opioids like sufentanil. Notably, similar reductions in opioid use were observed in ERAS implementations in a prospective study conducted by a community hospital, which demonstrated a significant decline in both the use and duration of patient-controlled analgesia, without increasing readmission or complication rates [5].

Our findings demonstrated that sufentanil use was significantly lower in the ERAS group compared to the control group, while resting and movement-related pain scores on postoperative day 1 were significantly lower in the ERAS group. Additionally, the proportion of participants requiring postoperative analgesics was markedly reduced in the ERAS group (Table 3). The lower pain levels despite reduced opioid use are likely attributable to the comprehensive measures implemented under the ERAS pathway, including reduced tourniquet use (2.6% in the ERAS group vs. 100% in the control group), preoperative dexamethasone administration, and layer-by-layer ropivacaine infiltration at the surgical site, which provided effective pain relief.

The time to independent ambulation was significantly shorter in the ERAS group, with participants walking unaided approximately 1.5 h earlier than those in the control group (5.02 ± 1.76 h vs. 6.30 ± 1.80 h, p < 0.001, Table 3). Early functional recovery is influenced by multiple factors, including PONV, pain, use of indwelling catheters or drains, and muscle strength. In this study, the ERAS pathway improved recovery by reducing thirst discomfort, minimizing PONV incidence, alleviating movement-related pain, and limiting the use of urinary catheters and surgical drains, thereby enhancing mobility. The use of short-acting general anesthetics also facilitated rapid and complete recovery of muscle strength [27].

ERAS fundamentally represents a multidisciplinary approach that optimizes clinical resources and improves perioperative management based on existing evidence and clinical practices. Key elements of the protocol include shortened preoperative fasting, reduced thirst discomfort, and PONV; use of short-acting anesthetics, intraoperative thermal insulation, and restrictive fluid management; and postoperative pain relief and early functional recovery. These measures collectively minimize surgical and anesthetic disruption of physiological functions, reducing the need for prolonged medical care and shortening the average hospital stay. In this study, the ERAS group achieved a significantly shorter average hospital stay (5.92 ± 1.16 days vs. 8.17 ± 1.76 days in the control group), representing a reduction of approximately 2 days. Notably, this reduction was primarily attributable to shorter postoperative stays (4.06 ± 1.09 days in the ERAS group vs. 6.06 ± 1.87 days in the control group, p < 0.001, Table 3). Furthermore, although a significant proportion of our study population consisted of elderly patients with chronic comorbidities such as hypertension and diabetes, the ERAS protocol remained effective in improving outcomes without increasing postoperative complications or 30 day readmission rates. This suggests that neither age nor comorbidity burden necessarily impairs the safety or effectiveness of ERAS. However, future studies should consider conducting subgroup analyses to assess whether tailored modifications to the ERAS protocol could further optimize recovery in specific populations, such as the frail elderly or patients with multiple comorbidities. In addition, further research could explore the applicability of ERAS protocols to other types of orthopedic procedures beyond joint arthroplasty, as well as investigate long-term outcomes such as functional recovery, quality of life, and healthcare utilization after discharge. These directions would enhance our understanding of how to personalize and expand the benefits of ERAS in broader clinical contexts.

## Limitations

One limitation of this study is the variability in standard perioperative practices across study centers, as the control group was managed according to local clinical routines. For instance, some centers routinely placed urinary and surgical drains, while others did not. Additionally, differences in anesthesia induction doses, intraoperative anesthetic techniques and fluid management strategies were observed. Such inter-center variability may have introduced heterogeneity in the outcomes of the control group, potentially affecting comparability with the ERAS group. Nonetheless, this variation also reflects real-world clinical practice, and the consistent trends observed in favor of the ERAS group across centers suggest that the benefits of the ERAS pathway are robust and generalizable, significantly reducing both average and postoperative hospital stays.

Another limitation is the potential for bias introduced by the gradual adoption of ERAS pathway by clinicians over the course of the study. For example, in the later stages, many clinicians began permitting clear liquids up to 2 h preoperatively and incorporating local infiltration anesthesia without concerns about wound healing, potentially underestimating the true effectiveness of the ERAS pathway. Future large-scale, real-world observational studies would be warranted to further elucidate the safety and efficacy of ERAS.

## Conclusion

The application of an ERAS pathway for primary TKA significantly reduces the length of hospital stay, alleviates postoperative pain, and lowers the incidence of adverse events compared to perioperative management without ERAS pathway implementation, thereby promoting accelerated recovery.

## Data Availability

The datasets used and/or analyzed during the current study are available from the corresponding author upon reasonable request. Individual participant data (IPD) and the study protocol will be accessed under request to Dr. Ren Liao. Data will become available from January 1, 2023, and will remain accessible for a period of 5 years.

## Abbreviations

ERAS: Enhanced recovery after surgery
TKA: Total knee arthroplasty
LOS: Length of hospital stay
PONV: Postoperative nausea and vomiting
NRS: Numerical rating scales
CRS: Central randomization system
BIS: Bispectral index
PCA: Patient-controlled analgesia
ITT: Intent-to-treat
ASA: American Society of Anesthesiologists
BMI: Body mass index
POCD: Potential postoperative cognitive dysfunction
IPD: Individual participant data
UIN: Unique identifying number

## Declarations

### Research registration unique identifying number (UIN)

(1) Name of the registry: The National Institutes of Health Clinical Trials Registry. (2) Unique identifying number or registration ID: NCT03517098. (3) Hyperlink to the registration (must be publicly accessible and will be checked): https://clinicaltrials.gov/study/NCT03517098?cond=NCT03517098&rank=1

### Guarantor

The corresponding author, Dr. Ren Liao, is the guarantor of this study and accept full responsibility for the work and/or the conduct of the study, had access to the data, and controlled the decision to publish.
